# The Consumption of *Lacticaseibacillus rhamnosus* HDB1258 Changes Human Gut Microbiota and Induces Immune Enhancement Through NK Cell Activation

**DOI:** 10.3390/microorganisms12102109

**Published:** 2024-10-21

**Authors:** Jin-Joo Lee, Kyung-Min Kim, Hyeon-Jeong Kim, Johann Sohn, Ji-Won Song, Hye-Yeon Koo, Seunghun Lee

**Affiliations:** 1 Biohealthcare R&D Center, Hyundai Bioland Co., Ltd., Manhae-ro, Danwon-gu, Ansan 15407, Republic of Korea; leejj@hyundaibioland.co.kr (J.-J.L.); khj1016@hyundaibioland.co.kr (H.-J.K.); isohn@hyundaibioland.co.kr (J.S.); izmsn@hyundaibioland.co.kr (J.-W.S.); 2 Department of Family Medicine, Seoul National University Bundang Hospital, Seongnam 13620, Republic of Korea; 3 Department of Family Medicine, Seoul National University College of Medicine, Seoul 03080, Republic of Korea

**Keywords:** probiotics, gut microbiota, immune enhancement, natural killer cell activity, *Lacticaseibacillus rhamnosus*

## Abstract

The gut microbiota can play an important role in enhancing the host’s complex immune system. In this regard, many studies indicate that probiotics consumption has a beneficial impact on alterations in the composition of the gut microbiota. Our previous study demonstrated that the oral administration of *Lacticaseibacillus rhamnosus* HDB1258 (HDB1258) enhances immune cell activity and alters the composition of gut microbiota in C57BL/6 mice, thereby showing its potential as a novel immunostimulatory ingredient. Therefore, this clinical trial assessed the effects of HDB1258 on human natural killer (NK) cell activity and changes in gut microbiota. It also investigated the correlation between gut microbiota and NK cell activity following HDB1258 supplementation. Participants (*n* = 71) were randomized into placebo and HDB1258 groups, and NK cell activity and gut microbiota were investigated at baseline (week 0) and endline (week 8). The present study showed that HDB1258 significantly increased NK cell activity and resulted in positive regulatory effects on the gut microbial balance in subjects compared to the placebo group. HDB1258 affected the gut microbial balance by inducing the growth of beneficial bacteria such as *Lactococcus* and *Sutterella*. Especially, the changes in *Escherichia*–*Shigella* composition were negatively correlated with the changes in NK cell activity after HDB1258 consumption. There was also a positive correlation between the NK cell activity in the HDB1258 group and the composition of *Prevotella* 9 and *Adlercreutzia*. These findings suggest that HDB1258 may improve the host’s intestinal environment by regulating gut bacteria related to immune response and promote NK cell activation. This study was registered at clinical research information service (CRIS: KCT0008204).

## 1. Introduction

Regulation of the gut microbial community has attracted attention as one of the key mechanisms for enhancing human immunity [1]. Commensal microbiota inhibit pathogen penetration by producing metabolites such as short-chain fatty acids (SCFAs), bile acids, or bacteriocins and competing for nutrients and adhesion to mucus layers or epithelial cells [2]. In addition, microbiota participate in the innate immune response by interacting directly with intestinal epithelial cells and immune cells, and a number of studies have reported that its dysbiosis is associated with immune system abnormalities [3,4]. Many studies have shown that the gut microbiota is an important indicator of host immune system. The intestinal microbial composition in patients with ulcerative colitis tended to have high levels of *Haemophilus* and *Olsenella*, and low levels of *Butyricimonas*, *Coprobacter*, and *Lactococcus* compared to healthy adults [5]. Furthermore, Gupta et al. [6] have reported that the group with alcoholic hepatitis had an increase in pathogens such as *Klebsiella pneumonia*, whereas ingestion of probiotics (*Lacticaseibacillus rhamnosus* R0011 and *Lactobacillus helveticus* R0052) was accompanied by a restoration of liver function through the regulation of their gut microbial imbalance by increasing the abundance of SCFAs producing bacteria. Furthermore, in patients with recurrent respiratory infections and inflammatory bowel syndrome, probiotics supplementation reduced their respective symptoms with increased levels of *Lactobacillus* and *Bifidobacterium* [7]. Li et al. [8] reported that the commensal gut bacteria can boost immune function by producing beneficial metabolites.

Probiotics, live microorganisms exhibiting supportive effects on host’s health, are generally known to exert a wide spectrum of health-beneficial effects, including protective activity against pathogenic infection and immunomodulatory efficacy through the gastrointestinal tract [9,10,11]. According to previous studies, several probiotics such as *L. rhamnosus* GG, *Lactobacillus gasseri* SBT2055, and *Bifidobacterium bifidum* PRL2010 exerted the immune-enhancing effects by the regulation of cytokines and the production of immunoglobulins [12,13,14]. Lee et al. [15] have demonstrated that *Weissella cibaria* JW15 enhances the activation of natural killer (NK) cells, a type of lymphocyte which plays a critical role for innate immunity, in nondiabetic subjects [16]. Similarly, it has been reported that intake of probiotics stimulated NK cell activity and improved innate immunity [17].

To date, several recent studies have provided results on the correlation between gut microbiota and changes in immune markers. For example, serum IL-1β and TNF-α were positively correlated with *Erysipelatoclostridium* and negatively correlated with *Enterococcus* and *Macrococcus* [18]. *Faecalibacterium* and *Ruminococcaceae* UCG-002 were positively correlated with CD4 + T cell, whereas a negative correlation was found between CD4/CD8 ratio and *Escherichia*–*Shigella* [19]. Notably, NK cells are associated with specific gut microbes or their metabolism. A previous clinical study reported a negative correlation between NK cell activity and *Ruminococcus* [20]. Metabolites such as gut-microbial-mediated N-acetyl-D-glucosamine or SCFAs have been shown to have host-protective effects against influenza, tumor cells through modulation of NK cell activity [21,22]. Nevertheless, the correlation between immune markers and gut microbiota in adults remains unclear. In order to understand the impact of probiotics on the complex correlations, it is necessary to provide a range of basic data at a clinical level.

*L. rhamnosus* HDB1258 (HDB1258) has exhibited remarkable mucin-binding and immunostimulatory activities by regulating immune cell activity in previous in vitro and in vivo studies [23,24]. Especially in healthy mice, HDB1258 administration enhanced innate immunity via activation of nuclear factor kappa B (NF-κB) signaling pathway, thereby stimulating NK cells and macrophages activity, as well as inducing pro-inflammatory cytokines and immunoglobulin A (IgA) levels. Furthermore, HDB1258-administered mice showed significant changes in gut microbiota composition compared to the untreated control mice. Based on these prior findings, the objectives of this study were twofold: (1) to evaluate the effect of HDB1258 on NK cell activity and gut microbiota in humans and (2) to investigate whether there is a significant correlation between gut microbiota and NK cell activity in the HDB1258 group compared to the placebo group. 

## 2. Materials and Methods

### 2.1. Clinical Subjects

**Figure 1:**
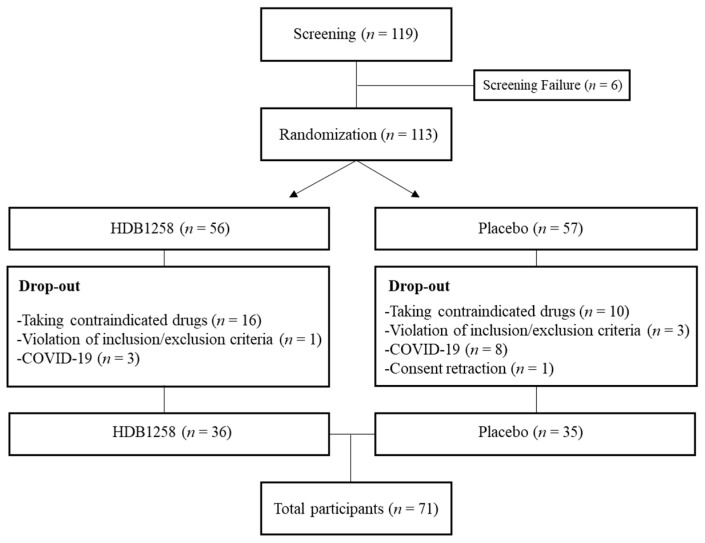
Participants flowchart of HDB1258 and placebo group.

This clinical study was designed as a randomized, double-blind, placebo-controlled trial of 8 weeks of HDB1258 supplementation. Among 119 volunteers aged 19 to 75 years, 71 who met the inclusion/exclusion criteria were finally randomized to the placebo (*n* = 35) and HDB1258 (*n* = 36) groups. The flow of the trial is shown in Figure 1. Participants voluntarily signed the consent form before study initiation, and the suitability of the following evaluation factors was investigated through visit evaluation: The inclusion criteria were those with a peripheral blood leukocyte count of 4.0 × 10^3^–10.0 × 10^3^ cells/μL and at least one episode of immunocompromised immunity, such as a herpes or cold, within 6 months to 1 year prior to screening. The exclusion criteria, taking into account participants’ health status and medications that may affect immune function, were as follows: constant consumption of any probiotics product or intake of medication or functional foods related to immunity within 2 weeks prior to screening; an allergy to probiotics; diabetes; a history/presence of significant disease, acute or chronic infection, or kidney, liver, gastrointestinal, or any other acute or chronic disease requiring treatment; pregnant or lactating woman; and have been vaccinated or diagnosed with COVID-19 within the last 2 months. Subjects taking any medications were also excluded. Detailed inclusion and exclusion criteria are listed in Supplementary Table S1.

This study followed the guidelines of the Declaration of Helsinki, and all procedures involving the human body were approved by the Institutional Review Board of Seoul National University Bundang Hospital (approval number B-2105-683-003). In addition, this study was registered at clinical research information service (CRIS), No. KCT0008204, on 17 February 2023.

### 2.2. Experimental Design

**Figure 2:**
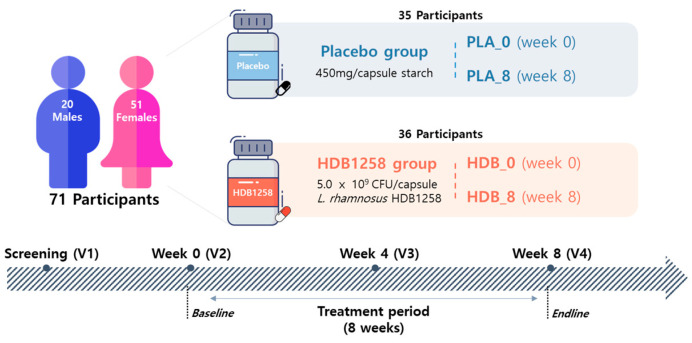
Experimental flowchart of the HDB1258 and placebo group.

The subjects were divided into placebo and HDB1258 groups (Figure 2). The HDB1258 group consumed 1 capsule (450 mg/capsule) that contained 5.0 × 10^9^ colony-forming units (CFUs) of HDB1258 per capsule once daily for 8 weeks, whereas the placebo group consumed 1 capsule that contained only starch as a control. To analyze the changes in the gut microbiota and NK cell activities induced by the consumption of HDB1258, fecal and blood samples were collected at baseline (week 0) and endline (week 8). Fecal samples were collected using an Accustool kit (AccuGene, Incheon, Republic of Korea), which was suspended in a preservative solution immediately after collection and sealed to prevent contamination and changes in fecal microbiota during storage. The collected fecal samples were stored at room temperature, and DNA was extracted and used for analysis within one month.

### 2.3. NK Cell Activity Measurement

To evaluate NK cell activity, venous blood of 71 selected subjects was collected and heparinized to isolate peripheral blood mononuclear cells (PBMCs) (effector cells) by density-gradient centrifugation. Human leukemia cell line K562 cells were used as target cells and co-cultured with PBMCs in effector-cells/target-cells ratios (E:T ratios) of 12.5:1 and 25:1. The NK cell activity was evaluated via a lactate dehydrogenase (LDH) assay using a CytoTox 96^®^ Non-Radioactive Cytotoxicity Assay Kit (Promega Co., Madison, WI, USA) and calculated by following the formula provided in the manufacturer’s instructions.

$$\mathrm{NK}\;\mathrm{cell}\;\mathrm{activity}\;(\%)=\frac{\left(\mathrm{Experimental}-\mathrm{Effector}\;\mathrm{Spontaneous}-\mathrm{Target}\;\mathrm{Spontaneous}\right)}{\left(\mathrm{Target}\;\mathrm{Maximum}-\mathrm{Target}\;\mathrm{Spontaneous}\right)}\times 100$$

### 2.4. DNA Extraction of Fecal Samples and Sequencing

DNA extraction from the fecal samples was performed in accordance with the manufacturer’s instructions using the AccuStool DNA Preparation Kit (AccuGene, Incheon, Republic of Korea). The hypervariable V4 region of the 16S rRNA gene was amplified through 25 PCR cycles using KAPA HiFi HotStart ReadyMix (Roche, Basel, Switzerland) and barcoded fusion primers 515fb/806rb containing Nextera adaptors (Illumina, San Diego, CA, USA). The amplified products (~250 bp) were purified with HiAccuBeads (AccuGene, Incheon, Republic of Korea) [25]. The amplicon libraries were pooled at an equimolar ratio, and the pooled libraries were sequenced on an Illumina MiSeq system using a MiSeq Reagent Kit v2 for 500 cycles (Illumina, San Diego, CA, USA).

### 2.5. Sequencing Data Preprocessing and Bioinformatics Analysis

For all raw datasets, VSEARCH v2.10.3 and the QIIME 1.9.1 software package (http://qiime.org accessed on 26 May 2015) were used to remove chimeric 16S rRNA gene sequences from filtered reads and to analyze downstream the quality-filtered chimera [26,27]. Each of the quality-filtered sequencing-read datasets was assigned to operational taxonomic units (OTUs) with a threshold of 97% pairwise identity using QIIME’s reference-based workflow scripts.

All sequencing result files (OTUs, Taxonomy, phylogenetic tree) were imported to R (v4.1.0), and a phyloseq object was generated using the phyloseq package for microbiota analysis [28]. Microbial composition was analyzed at the phylum, family, and genus levels, and the significantly changed microbiota compared to placebo during the intervention period was analyzed. Alpha diversity is represented by using Observed species, Shannon, and Chao1 indices, and beta diversity is represented by using a principal coordinate analysis (PCoA) plot based on a Bray–Curtis dissimilarity index. Linear discriminant analysis effect size (LEfSe) was performed to identify specific microorganisms that exhibited differential abundance of each group using the MicrobiomeMarker package [29]. Pearson’s correlation was performed to determine the correlation between the fecal microbiota and NK cell activity. All visualizations were performed using the ggplot2 package of R [30].

### 2.6. Statistical Analysis

All data were expressed in mean or median values and standard deviation (SD). A Shapiro test in R was conducted for the normality test, and a *t*-test or Wilcoxon test was chosen depending on normality (normality; *t*-test or paired *t*-test, non-normality; Wilcoxon signed-rank sum test or Mann–Whitney U test)**.** The significance according to the intervention period in the group was analyzed with the paired *t*-test or Wilcoxon signed-rank sum test. In addition, the significance between the groups was performed using the *t*-test or Mann–Whitney U test. PERMANOVA was used to analyze significant differences in beta diversity between groups. For the LEfSe analysis, the significance threshold of the Kruskal–Wallis (KW) test was set at 0.05 and a value of 2.0 for the linear discriminant analysis (LDA) score threshold. The Pearson’s correlation coefficient was analyzed using the microbiome package of R program at the genus level. The statistically significant difference was considered to be *p* < 0.05.

### 2.7. Nucleotide Sequence Accession Numbers

The sequencing data of 16S rRNA genes are publicly available in the NCBI Short Read Archive under accession number SRP506441 (NCBI BioProject PRJNA1109304).

## 3. Results

### 3.1. Effects of HDB1258 on NK Cell Activity

**Figure 3:**
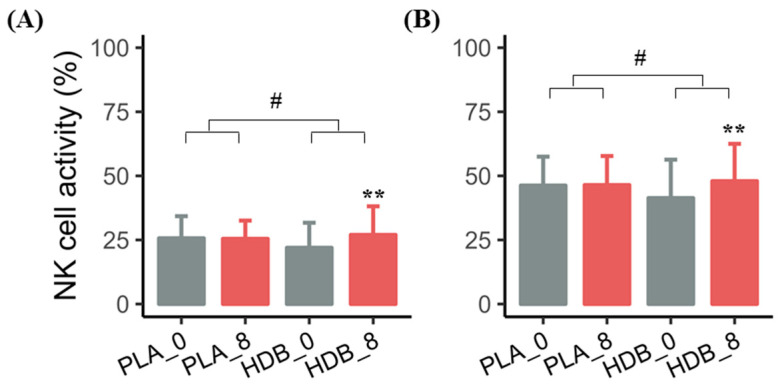
Bar plot for NK cell activity E:T (%) changes before and after the 8-week supplementation in each group. (**A**) NK activity: 12.5:1, (**B**) NK activity: 25:1; Mean ± SD, ** *p* < 0.01, *p*-value for paired *t*-test or Wilcoxon signed-rank test compared within groups; # *p* < 0.05, *p*-value for two sample *t*-test or Wilcoxon rank sum test compared between groups for changed value. PLA_0: baseline in placebo group, PLA_8: endline in placebo group, HDB_0: baseline in HDB group, HDB_8: endline in HDB group.

NK cell activities in the E:T ratios of 12.5:1 and 25:1 were significantly increased by 5.03 ± 9.16% and 6.60 ± 12.11%, respectively, in the HDB1258 group (*p* < 0.01), while no significant changes were observed in the placebo group in the comparison before and after intake (Table 1 and Figure 3). Moreover, NK cell activities in overall tested E:T ratios also showed remarkable induction compared to the placebo group (*p* < 0.05).

### 3.2. Microbial Diversity Within and Between Placebo and HDB1258 Groups

**Figure 4:**
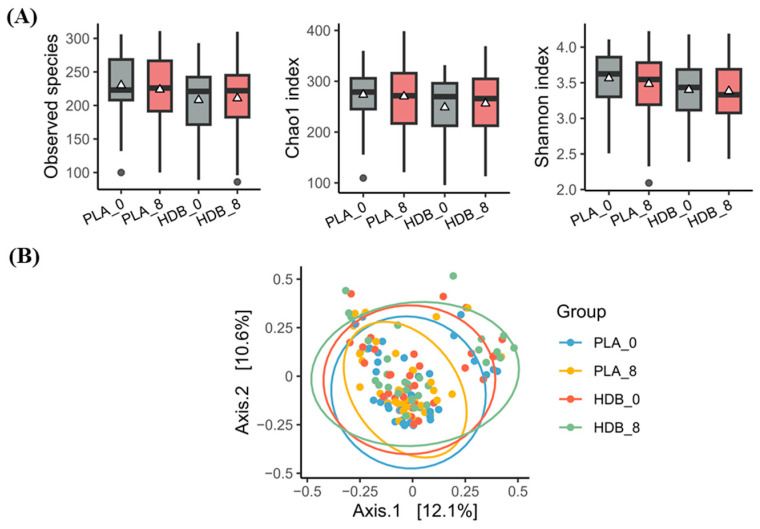
Diversity comparisons of the gut microbiota before and after supplementation. (**A**) Alpha diversity based on Observed species, Shannon, Chao1. Box plot shows median value as well as interquartile range (IQR). *p*-value for paired *t*-test or Wilcoxon signed-rank test compared within groups. △; mean value. (**B**) Beta diversity on PCoA analysis of Bray–Curtis distances. Significance is compared between groups using PERMANOVA. PLA_0: baseline in placebo group, PLA_8: endline in placebo group, HDB_0: baseline in HDB group, HDB_8: endline in HDB group.

In this study, alpha diversity was evaluated with three indicators—Observed species, Shannon index, and Chao1 index (Figure 4A). No notable alterations in alpha diversity were discerned in either the HDB1258 or placebo groups subsequent to ingestion. Beta diversity analysis to compare microbial diversity between groups also showed no significant differences (Figure 4B).

### 3.3. Effects of HDB1258 on Composition of Gut Microbiota

**Figure 5:**
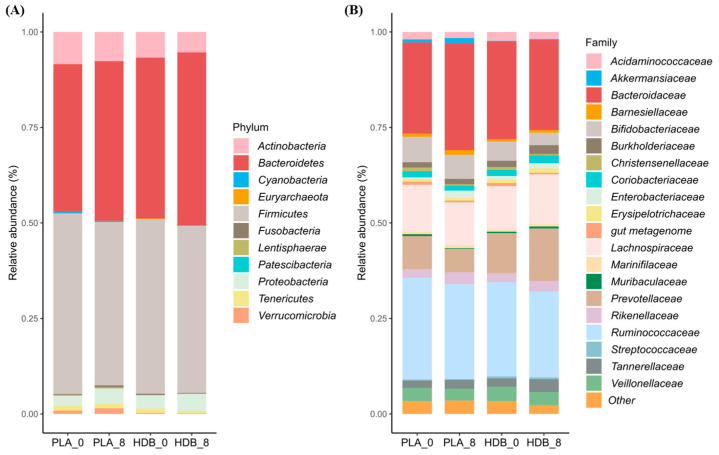
Stacked bar of relative abundance (%) at (**A**) phylum and (**B**) family level (family level shows only top 20 bacteria). PLA_0: baseline in placebo group, PLA_8: endline in placebo group, HDB_0: baseline in HDB group, HDB_8: endline in HDB group.

**Figure 6:**
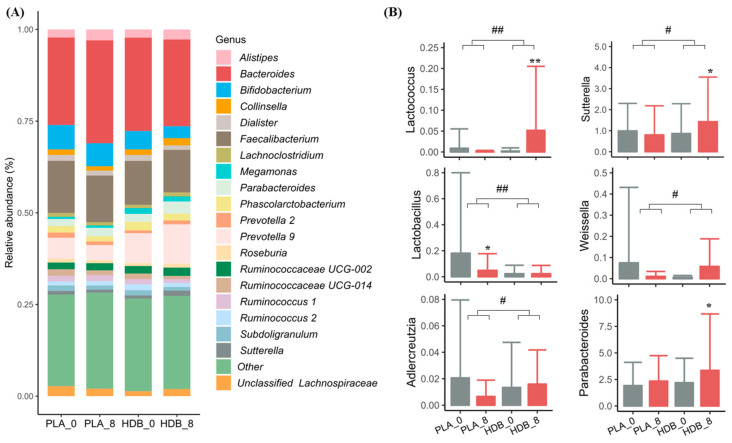
Comparison of gut microbiota composition following HDB1258 intervention in each group. (**A**) Stacked bar of relative abundance (%) at genus level (show only top 20 bacteria) (**B**) Comparison of relative abundance (%) after ingestion in the group compared to baseline. * *p* < 0.05, ** *p* < 0.01, *p*-value for paired *t*-test or Wilcoxon signed-rank test compared within groups; # *p* < 0.05, ## *p* < 0.01, *p*-value for two sample *t*-test or Wilcoxon rank sum test compared between groups for changed value. PLA_0: baseline in placebo group, PLA_8: endline in Placebo group, HDB_0: baseline in HDB group, HDB_8: endline in HDB group.

HDB1258 group showed changes in gut microbial composition at the phylum and family levels compared to the placebo group (Figure 5). As shown in Table 2, phylum *Firmicutes* showed a significant decrease only in the placebo group (*p* < 0.05). Family *Burkholderiaceae* and *Tannerellaceae* significantly increased and *Bifidobacteriaceae* decreased in the HDB1258 group (*p* < 0.05). In the comparison of abundance changes, significant differences were found between the placebo and HDB1258 groups. In particular, *Bacteroidaceae* and *Lactobacillaceae* were significantly increased and decreased, respectively, in the placebo group, but showed no changes in abundance in the HDB1258 group (*p* < 0.05 and *p* < 0.01). The relative abundance of the top 20 at the genus level is shown in Figure 6A. Specifically, in Figure 6B, a significant increase in *Lactococcus*, *Sutterella*, and *Parabacteroides* were found in the HDB1258 group compared to placebo group. In addition, some genera showed significant differences in the amount of abundance change before and after ingestion between the two groups; *Lactococcus*, *Sutterella*, *Adlercreutzia*, and *Weissella* tended to increase in contrast to the placebo group. On the other hand, *Lactobacillus* showed a significant decrease only in the placebo group, and no such observation was found in the HDB1258 group (*p* < 0.05). Detailed data are shown in the Supplementary Materials (Tables S2–S4).

### 3.4. LEfSe Analysis

**Figure 7:**
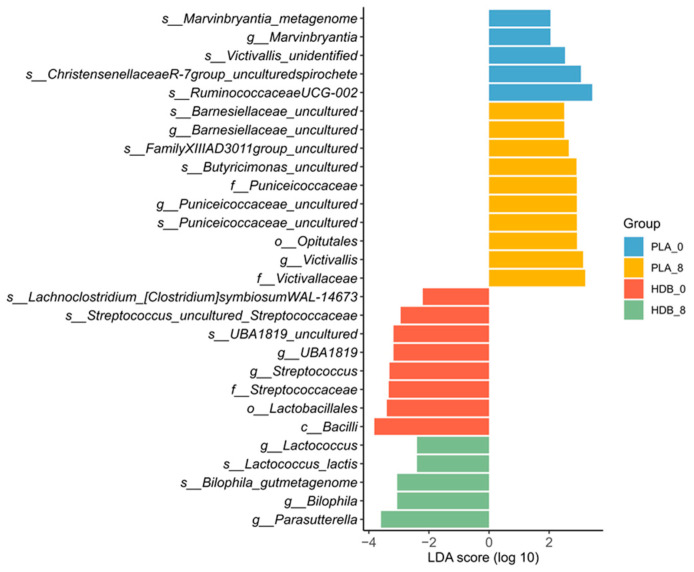
Microbiota biomarkers with significant abundance in each group (*p* < 0.05, LDA score > 2.0). Taxonomic level; *c* = class, *o* = order, *f* = family, *g* = genus, *s* = species). Negative direction: bacteria showing significant abundance at baseline and endline in the HDB1258 group. Positive direction: bacteria showing significant abundance at baseline and endline in the placebo group. PLA_0: baseline in placebo group, PLA_8: endline in placebo group, HDB_0: baseline in HDB group, HDB_8: endline in HDB group.

LEfSe analysis was performed to identify differentially abundant biomarker taxa at each time point in the placebo and HDB1258 groups. Family *Victivallaceae* and *Puniceicoccaceae* showed significantly enriched after placebo ingestion, while genus *Lactococcus*, *Parasutterella*, and *Bilophila* were significantly higher after HDB1258 ingestion (Figure 7).

### 3.5. Correlation Between NK Cell Activity and Gut Microbiota

**Figure 8:**
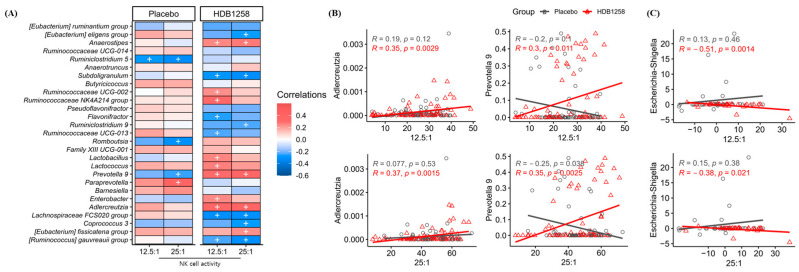
Pearson’s correlation analysis between gut microbiota and NK cell activity (%): (**A**) correlation heatmap with NK cell activity (%). Red and blue colors represent positive and negative correlations. + *p* < 0.05; (**B**) scatter plots with Pearson’s correlation coefficients between relative abundance of gut microbiota (%) and NK cell activity (12.5:1 and 25:1); (**C**) changes in relative abundance of gut microbiota and NK cell activity (%) in each group.

Pearson’s correlation analysis was conducted to investigate the correlation between fecal microbiota and NK cell activity. Four genera in the placebo group and seventeen genera in the HDB1258 group showed an association between NK cell activities and their abundance (Figure 8A). Especially, *Adlercreutzia* (NK12.5:1, r = 0.35, *p* = 0.0029; NK25:1, r = 0.37, *p* = 0.0015) and *Prevotella* 9 (NK12.5:1, r = 0.30, *p* = 0.011; NK25:1, r = 0.35, *p* = 0.0025) were positively correlated with all ratios (12.5:1 and 25:1) of NK cell activity increased by HDB1258 (Figure 8B). In contrast, *Escherichia*–*Shigella* (NK12.5:1, r = −0.51, *p* = 0.0014; NK25:1, r = −0.38, *p* = 0.021) showed significant negative correlation between the changes in its abundance and NK cell activity (Figure 8C).

## 4. Discussion

In a placebo-controlled clinical study in 71 adults, HDB1258 significantly enhanced NK cell activity and was shown to improve the gut microbiota by increasing the composition of beneficial gut bacteria. There was a marked increase in beneficial bacteria such as *Lactococcus* and *Sutterella* in humans. We also confirmed a correlation between the changes in gut microbes and NK cell activity in the HDB1258 group. Likewise, in the mouse model [24], HDB1258 enhanced NK cell activity along with an increase in IgA and TNF-α. In the analysis of fecal microbiota from mice, significant changes in microbial composition were observed in response to HDB1258 treatment compared to the placebo group (FJ880724_s, PAC001072_s). Additionally, PAC001765_g and PAC002480_g were found to correlate with an increase in the TNF-α/IL-10 ratio; these taxa were identified exclusively in mice. Given the considerable differences in gut microbiota composition between mice and humans, the effects may vary across models [31]. Nevertheless, HDB1258 demonstrated the ability to enhance the activity of immune markers in both mouse and human models, thereby inducing changes in gut microbiota and confirming its potential as a functional material for immune enhancement.

NK cells play important roles in the maintenance of immune homeostasis by leading defective and infected cells to apoptotic or necrotic cell death, meaning the removal of dysfunctional cells [32,33]. As NK cells contribute to both activation of innate immunity and formation of adaptive immunity, their activity is widely used as a key biomarker in the evaluation of host’s immunity [34]. In this study, the HDB1258 group showed significantly increased NK cell activity, and it was probably assumed that stimulated dendritic cells (DCs) promoted NK cell activation. Previous research has demonstrated that probiotics have regulatory effects on DCs, potent stimulators of NK cells [35]. Also, many studies have suggested that *L. rhamnosus* may contribute to T cell activation by modulating immune function of DCs and subsequently exhibit regulatory effects on Th1- and Th2-immune responses [36,37]. Therefore, increased NK cell activity is expected to support the development of adaptive immunity, including the strengthening of innate immunity.

*L. rhamnosus* has demonstrated clinical effects in human studies. The consumption of *B. animalis* subsp. *lactis* HN019 and *L. rhamnosus* HN001 showed a constipation-relieving effect, associated with a decrease in UCG-002 and *Eisenbergiella*, along with an increase in *Anaerostipes* [38]. Additionally, *L. rhamnosus* Probio-M9 demonstrated an improvement in stress levels in adults, resulting in increased levels of *Barnesiella* and *Akkermansia* [39]. Despite the demonstrated beneficial effects of *L. rhamnosus* in humans, it remains crucial to establish the clinical efficacy of specific probiotics. Interestingly, previous studies [40,41] demonstrated that the composition of gut microbes was associated with enhanced NK cell activity. For example, maturation of liver-resident NK cells in antibiotic-treated mice was inhibited with a decrease in the abundance of beneficial microbiota species, classified as genus *Akkermansia*, family *Prevotellaceae*, *Ruminococcaceae*, and *Lachnospiraceae* [40]. Furthermore, activation of NK cells by postbiotics mixtures in immunosuppressed mice was linked to the regulation of gut anaerobic bacteria such as *Bifidobacteria*, *Lachnospiraceae*, and *Lactobacillaceae* [41]. From this perspective, we observed changes in the gut microbiota following HDB1258 supplementation and confirmed a correlation between increased NK cell activity and the abundance of specific gut microbes. In particular, our investigation was more notable for analyzing the effects of HDB1258 on more diverse gut microbiota changes at the genus level.

In the HDB1258 group, the abundance of *Bifidobacteriaceae* at the family level showed a significant decrease; however, no significant difference was observed in the change compared to the placebo group (*p* = 0.8315; Table 2). Generally, *Bifidobacterium*, which belongs to the *Bifidobacteriaceae* family, known for its probiotics properties, is recognized for its benefits to host health. However, a previous clinical study reported a decrease in *Bifidobacterium* with the intake of *Lactobacillus* species. Considering the characteristics of *Bifidobacterium* and *Lactobacillaceae*, which primarily colonize the upper part of the intestine, the intestinal enrichment of *Lactobacillaceae* may be related to the colonization of *Bifidobacterium* [42]. The genus level was narrowed down to five genera (*Adlercreutzia*, *Prevotella*, *Lactococcus*, *Sutterella*, and *Escherichia*–*Shigella*). These genera were reported to directly or indirectly stimulate specific immune-signaling pathways or immune cells within the host or produce useful metabolites. Of note, HDB1258 intake showed a significant increase in *Lactococcus* and *Sutterella*. The genus *Sutterella* is the dominant bacteria of the duodenum and may exhibit mild levels of inflammatory activity in the intestinal tract. This activity was significantly lower than the excessive inflammatory response caused by pathogens, so the authors hypothesized that *Sutterella* would lead to an activating effect on immune activity [43]. Similar to our findings, Korpela K et al. [44] reported that *L. rhamnosus* GG supplementation was associated with a significant increase in *Lactococcus*. *Lactococcus* is known as representative probiotics to produce bacteriocins [45]. It has been previously reported that some species of *Lactococcus* exerts immune-enhancing effects via activation of NK cells and modulation of the pivotal signaling pathways (e.g., mitogen-activated protein kinase (MAPK) and NF-κB) that are essential for regulating the immune response [46,47,48]. HDB1258 was observed to activate macrophages and NK cells through NF-κB signaling in healthy mice [24]. The results suggest that HDB1258 strain itself is functional by stimulating immune cells in the large intestine, and the ingestion of HDB1258 also enhances immune function by inducing changes in the gut microbiota.

From this analysis, it was assumed that increased NK cell activity after HDB1258 intervention would correlate with specific gut microbiota abundance. In the HDB1258 group, *Adlercreutzia*, *Prevotella* 9, and *Escherichia*–*Shigella* were found to be significantly correlated with increased NK cell activity. *Adlercreutzia* is known to produce SCFAs and equol by metabolizing phytoestrogen, and its metabolites have been reported to have immunomodulatory properties [49]. Some species of *Prevotella* are known as endogenous airway microbiota and employ a defensive mechanism by inducing an innate immune response against the invasion of pathogens that can cause pneumonia [50]. Previous studies have shown that enrichment of SCFAs-producing bacteria may indicate immune improvement through reduction in pro-inflammatory cytokines [51]. Probably, the positive correlation between *Adlercreutzia* or *Prevotella* 9 and NK cell activity may be related to SCFAs-producing properties. On the other hand, *Escherichia*–*Shigella* are well-known opportunistic pathogens in the intestines that increase the risk of intestinal destruction and host infection and can cause excessive inflammation and diarrhea [52,53]. This genus showed excessive expansion with dysfunctional inflammatory responses in patients with IgA nephropathy [54]. Despite the inherent difficulty in identifying direct mechanisms of action between these gut microbiota and specific biomarkers, it is encouraging to see a statistically significant negative correlation. Consequently, we can only speculate that the observed correlation may be indirect, for example, through the modulation of immune responses by the production of metabolites such as SCFAs or links with gut immune cells. Also, according to in vitro studies, HDB1258 exhibited a competitive inhibitory effect on the opportunistic growth of enteric pathogens due to its exceptional acid and bile resistance and high adhesion to the mucin layer [23]. In a mice model of *Klebsiella-oxytoca*-induced colitis, HDB1258 ingestion was observed to significantly reduce colonic and systemic inflammatory markers [55]. Taken together, these results are expected to play a role in HDB1258 competitively excluding infectious pathogens from the outside, as well as opportunistic pathogens in the gut.

In the current study, the results showed that HDB1258 enhanced NK cell activity and altered gut microbiota such as *Lactococcus* and *Sutterella*. We have also found specific bacteria that correlate with increased NK cell activity. Taken together, HDB1258 may help improve host immunity by inducing changes in specific microbial populations and strengthening NK cell activity, suggesting that HDB1258 has a potential as a health-beneficial ingredient to enhance immune functions. However, to elucidate the mechanism of interaction between NK cells and gut microbiota, further studies are required to analyze the effects of changes in gut metabolite concentrations or HDB1258 colonization on the microbiota from multiple perspectives. To the best of our knowledge, there have been few studies that have analyzed the correlation between NK cell activity and gut microbiota in the context of immune enhancement studies. It is our hope that the results of this study will serve as a foundation for future research on the relationship between probiotics immune function and gut microbiota.

## 5. Conclusions

Our research builds on previous in vitro and in vivo studies to elucidate the effects of HDB1258 on the activation of immune cells in adults and the modulation of various gut microbiota. Remarkably, HDB1258 was found to induce the activation of NK cells, thereby enhancing innate immunity, while promoting the colonization of gut bacteria involved in immune response, particularly *Lactococcus* and *Sutterella*. Furthermore, the increased NK cell activity induced by HDB1258 was correlated with specific microbiota, including *Prevotella* 9, *Adlercreutzia*, and *Escherichia*–*Shigella*. These findings suggest that HDB1258 has the potential to create a beneficial gut environment in the host and contribute to immune enhancement.

## Figures and Tables

**Table 1:** Changes in participants’ NK cell activity (%) following supplementation.

E:T Ratio	Placebo (*n* = 35)			HDB1258 (*n* = 36)			
	Baseline	Endline	*p^a^*	Baseline	Endline	*p^a^*	*p^b^*
12.5:1	25.66 ± 8.61	25.46 ± 7.10	0.9095	21.94 ± 9.76	26.97 ± 11.16	<0.01	<0.05
25:1	46.23 ± 11.27	46.42 ± 11.32	0.9256	41.35 ± 14.99	47.95 ± 14.55	<0.01	<0.05

Baseline, Week 0; Endline, Week 8. *p^a^*, *p*-value for paired *t*-test or Wilcoxon signed-rank test compared within group; *p^b^*, *p*-value for two-sample *t*-test or Wilcoxon rank sum test compared between groups for changed value.

**Table 2:** Comparisons of relative abundance (%) at phylum and family level.

	Placebo				HDB1258				*p^c^*
	Baseline	Endline	*p^a^*	ΔChanges in Relative Abundance	Baseline	Endline	*p^b^*	ΔChanges in Relative Abundance	
	Phylum								
*Actinobacteria*	8.42 ± 8.14	7.64 ± 8.10	0.2286	−0.78 ± 9.080	6.72 ± 7.04	5.34 ± 9.070	0.05047	−1.38 ± 8.30	0.7693
*Bacteroidetes*	38.69 ± 16.74	41.77 ± 16.94	0.1067	3.084 ± 16.59	42.10 ± 15.58	45.34 ± 15.88	0.1376	3.24 ± 18.61	0.8948
*Firmicutes*	47.29 ± 13.36	42.79 ± 13.81	<0.05	−4.51 ± 12.50	45.73 ± 13.68	43.77 ± 14.39	0.1462	−1.97 ± 15.73	0.9587
*Proteobacteria*	2.65 ± 2.35	3.99 ± 6.32	0.5720	1.34 ± 6.23	3.48 ± 3.56	4.46 ± 5.57	0.2294	0.98 ± 5.06	0.7000
	Family								
*Bacteroidaceae*	23.85 ± 16.79	28.05 ± 17.36	<0.05	4.20 ± 11.21	25.45 ± 13.38	23.66 ± 14.34	0.4461	−1.79 ± 14.93	<0.05
*Bifidobacteriaceae*	6.68 ± 7.038	6.30 ± 7.46	0.2183	−0.38 ± 8.10	5.040 ± 6.27	3.27 ± 4.44	<0.05	−1.77 ± 5.53	0.8315
*Burkholderiaceae*	1.39 ± 1.47	1.41 ± 1.89	0.6740	0.022 ± 1.55	1.66 ± 1.57	2.33 ± 2.078	<0.05	0.67 ± 1.77	0.1333
*Rikenellaceae*	2.28 ± 2.19	3.075 ± 2.78	<0.05	0.79 ± 2.47	2.32 ± 4.58	2.82 ± 3.71	0.1157	0.50 ± 4.83	0.6496
*Tannerellaceae*	1.92 ± 2.20	2.34 ± 2.40	0.3298	0.43 ± 2.32	2.20 ± 2.30	3.35 ± 5.32	<0.05	1.16 ± 4.50	0.5384
*Lactobacillaceae*	0.18 ± 0.62	0.049 ± 0.13	<0.05	−0.13 ± 0.51	0.023 ± 0.066	0.024 ± 0.064	0.2425	0.00047 ± 0.048	<0.01

Baseline, Week 0; Endline, Week 8. *p^a^*, *p*-value for paired *t*-test or Wilcoxon signed-rank test compared within placebo group; *p^b^*, *p*-value for paired *t*-test or Wilcoxon signed-rank test compared within HDB1258 group; *p^c^*, *p*-value for two sample *t*-test or Wilcoxon rank sum test compared between groups for changed value.

## Data Availability

The sequencing datasets are publicly available in the NCBI Short Read Archive under accession number SRP506441, NCBI BioProject PRJNA1109304.

## Supplementary Materials

The following supporting information can be downloaded at: https://www.mdpi.com/article/10.3390/microorganisms12102109/s1, Table S1: Detailed included and excluded criteria; Table S2: Comparisons of relative abundance (%) at phylum level; Table S3: Comparisons of relative abundance (%) at family level (show top 10 bacteria and *Lactobacillaceae*); Table S4: Comparisons of relative abundance (%) at genus level.

## Abbreviations

NK: natural killer; SCFAs: short-chain fatty acids; NF- κB: nuclear factor kappa B; IgA: immunoglobulin A; CFU: colony forming unit; PBMCs: peripheral blood mononuclear cells; OTUs: operational taxonomic units; PCoA: principal coordinate analysis; LEfSe: linear discriminant analysis effect size; KW: Kruskal–Wallis; SD: standard deviation; LDA: linear discriminant analysis DCs: dendritic cells; MAPK: mitogen-activated protein kinase.
