# Supplementary material for: The Consumption of Lacticaseibacillus rhamnosus HDB1258 Changes Human Gut Microbiota and Induces Immune Enhancement Through NK Cell Activation
Source: Microorganisms. 2024 Oct 21;12(10):2109. doi: 10.3390/microorganisms12102109 (PMC11510592; doi:10.3390/microorganisms12102109)
Supplement: Supplementary file 1 [file microorganisms-12-02109-s001.zip › microorganisms-3220746-supplementary.pdf]

Supplementary Material

1 Supplementary Tables

Table S1. Detailed included and excluded criteria.

Included criteria	Excluded criteria
(1) Those with a peripheral blood leukocyte count of 4 x 10^3 cells/μL or more and less than 10 x 10^3 cells/μL measured in V1.	(1) Those who are currently being treated for severe cardiovascular, immune, respiratory, nervous, infectious diseases, etc.
(2) Based on V1, those who falls under one or more of the following criteria:<br>- Those who had upper respiratory tract infection (cold, acute tonsillitis, pharyngitis, etc.) (more than 2 times).<br>- Stomatitis (more than 2 times).<br>- Herpes zoster (shingles).<br>- Simple cystitis (more than 3 times) within 1 years.<br>- Those who had simple cystitis (more than 2 times) within 6 months.<br>- Those with a bowel leak syndrome questionnaire score of 4 or more were included.	(2) Systolic blood pressure greater than 160 mmHG or greater than 100 mmHg diastolic blood pressure.<br>(3) Diabetic patients with fasting blood sugar greater than 126 mg/dL, glycated hemoglobin (HbAlc) greater than 6.5%, or who are receiving antidiabetics (oral hypoglycemic agents, insulin, etc.).<br>(4) TSH is 0.1μIU/mL or less or 10μIU/mL or greater.<br>(5) Where vaccination is performed within two months based on V1.
	(6) Within 2 months based on V1, the COVID-19 confirmed patient.
	(7) If the AST (GOT) or ALT (GPT) is more than three times the upper limit of the normal operator.
	(8) If the creatine is at least 1.5 times the normal upper limit of the implementation agency.
	(9) Taking steroids within 4 weeks based on V1.
	(10) Administration of antibiotics, immunosuppressants, gastric acid inhibitors, and antihistamines within 2 weeks based on V1.
	(11) Intake of probiotics (more than 4 times a week) or immune-related health functional foods and vitamins within 2 weeks based on V1.
	(12) As a result of the drinking habits survey, if the drinking habits are more than 14 units (men) or 7 units (women) per week on average.
	(13) Severe allergies, treated with asthma.
	(14) Acute food allergy experience.
	(15) Severe gastrointestinal symptoms such as heartburn and indigestion.
	(16) Pregnant, lactating woman or If you have a pregnancy plan for the exam period.

(17) If you have participated in another clinical trial within 8 weeks of V1 or plan to participate in another clinical trial.

(18) If you are sensitive or allergic to food ingredients for human application testing.

(19) If your body mass index (BMI) is over 30kg/m^2.

Table S2. Comparisons of relative abundance (%) at phylum level.

Phylum	Placebo				HDB1258				p^c
	Baseline	Endline	p^a	ΔChanges in relative abundance	Baseline	Endline	p^b	ΔChanges in relative abundance	
Actinobacteria	8.42±8.14	7.64±8.10	0.2286	-0.78±9.080	6.72±7.04	5.34±9.070	0.05047	-1.38±8.30	0.7693
Bacteroidetes	38.69±16.74	41.77±16.94	0.1067	3.084±16.59	42.10±15.58	45.34±15.88	0.1376	3.24±18.61	0.8948
Cyanobacteria	0.38±1.61	0.30±1.24	0.3613	-0.085±0.39	0.00015±0.00088	0.00044±0.0020	0.7893	0.00030±0.0022	0.2569
Euryarchaeota	0.019±0.081	0.0055±0.018	0.3613	-0.013±0.072	0.14±0.85	0.059±0.34	0.3711	-0.085±0.51	0.9905
Firmicutes	47.29±13.36	42.79±13.81	<0.05	-4.51±12.50	45.73±13.68	43.77±14.39	0.1462	-1.97±15.73	0.9587
Fusobacteria	0.24±1.37	0.50±2.94	0.5839	0.27±1.58	0.37±1.88	0.20±0.87	0.4148	-0.17±1.041	0.2931
Lentisphaerae	0.17±0.39	0.31±1.17	0.9321	0.14±1.016	0.0089±0.030	0.0077±0.022	1.0000	-0.0012±0.034	0.9728
Proteobacteria	2.65±2.35	3.99±6.32	0.5720	1.34±6.23	3.48±3.56	4.46±5.57	0.2294	0.98±5.06	0.7000
Patescibacteria	0.0052±0.0097	0.0052±0.0099	0.6726	0.000072±0.010	0.0071±0.020	0.0074±0.026	0.5421	0.00030±0.020	0.5708
Tenericutes	1.23±3.41	1.19±3.43	0.5590	-0.034±4.21	1.16±3.51	0.64±1.48	0.6982	-0.51±3.59	0.2942
Verrucomicrobia	0.89±2.038	1.48±3.96	0.3696	0.59±4.27	0.27±0.83	0.16±0.41	0.6435	-0.10±0.92	0.1200

Baseline, Week 0; Endline, Week 8. p^a, *p*-value for paired t-test or Wilcoxon signed rank test compared within placebo group; p^b, *p*-value for paired t-test or Wilcoxon signed rank test compared within HDB1258 group; p^c, *p*-value for two sample *t*-test or Wilcoxon rank sum test compared between groups for changed value.

Table S3. Comparisons of relative abundance (%) at family level (show top 10 bacteria and *Lactobacillaceae*).

Family	Placebo				HDB1258				p^c
	Baseline	Endline	p^a	ΔChanges in relative abundance	Baseline	Endline	p^b	ΔChanges in relative abundance	
Acidaminococcaceae	1.91±2.92	1.56±2.19	0.09876	-0.35±1.45	2.32±3.63	1.85±2.67	0.5593	-0.47±3.42	0.5246
Bacteroidaceae	23.85±16.79	28.05±17.36	<0.05	4.20±11.21	25.45±13.38	23.66±14.34	0.4461	-1.79±14.93	<0.05
Bifidobacteriaceae	6.68±7.038	6.30±7.46	0.2183	-0.38±8.10	5.040±6.27	3.27±4.44	<0.05	-1.77±5.53	0.8315
Burkholderiaceae	1.39±1.47	1.41±1.89	0.6740	0.022±1.55	1.66±1.57	2.33±2.078	<0.05	0.67±1.77	0.1333
Lachnospiraceae	12.27±5.80	11.19±5.44	0.3881	-1.079±7.30	11.39±5.42	13.070±5.67	0.1770	1.68±7.32	0.1161
Prevotellaceae	8.64±15.18	6.15±11.98	0.5554	-2.50±10.70	10.53±15.12	13.72±19.40	0.1950	3.19±16.21	0.1800
Rikenellaceae	2.28±2.19	3.075±2.78	<0.05	0.79±2.47	2.32±4.58	2.82±3.71	0.1157	0.50±4.83	0.6496
Ruminococcaceae	26.63±11.31	24.92±12.44	0.2179	-1.72±8.09	24.67±14.82	22.47±12.92	0.3515	-2.20±13.99	0.8581
Tannerellaceae	1.92±2.20	2.34±2.40	0.3298	0.43±2.32	2.20±2.30	3.35±5.32	<0.05	1.16±4.50	0.5384
Veillonellaceae	3.46±3.84	3.073±4.22	0.3298	0.39±4.11	3.76±5.31	3.52±4.10	0.6887	-0.25±4.66	0.6663
Lactobacillaceae	0.18±0.62	0.049±0.13	<0.05	-0.13±0.51	0.023±0.066	0.024±0.064	0.2425	0.00047±0.048	<0.01

Baseline, Week 0; Endline, Week 8. p^a, *p*-value for paired t-test or Wilcoxon signed rank test compared within placebo group; p^b, *p*-value for paired t-test or Wilcoxon signed rank test compared within HDB1258 group; p^c, *p*-value for two sample *t*-test or Wilcoxon rank sum test compared between groups for changed value.

Table S4. Comparisons of relative abundance (%) at genus level.

Phylum	Genus	Placebo				HDB1258				p^c
		Baseline	Endline	p^a	ΔChanges in relative abundance	Baseline	Endline	p^b	ΔChanges in relative abundance	
Actinobacteria	*Adlercreutzia*	0.021±0.059	0.0066±0.012	0.08389	-0.014±0.061	0.013±0.034	0.016±0.026	0.1650	0.0026±0.022	<0.05
	Collinsella	1.48±2.61	1.18±1.53	0.8370	-0.30±2.25	1.50±2.087	1.94±5.39	0.3991	0.44±4.60	0.2198
Bacteroidetes	*Bacteroides*	23.85±16.79	28.05±17.36	<0.05	4.20±11.21	25.45±13.38	23.66±14.34	0.4461	-1.79±14.93	0.05995
	Paraprevotella	0.60±1.24	0.68±1.34	0.7764	0.085±1.43	1.0047±1.79	0.48±0.96	<0.01	-0.52±1.25	0.07885
	Prevotella 2	1.41±3.41	1.0050±3.30	0.1475	-0.41±1.98	0.77±2.94	1.057±5.087	0.9063	0.28±2.51	0.2713
	Prevotella 7	0.24±1.42	0.013±0.079	1.0000	-0.23±1.34	0.11±0.48	0.18±1.091	1.0000	0.074±0.70	0.5637
	Prevotella 9	5.72±12.42	4.084±10.47	0.8110	-1.63±8.92	8.16±14.09	10.72±17.66	0.2069	2.56±14.12	0.2846
	Parabacteroides	1.92±2.20	2.34±2.40	0.3298	0.43±2.32	2.20±2.30	3.35±5.32	<0.05	1.16±4.50	0.5384
Firmicutes	*Thermoanaero-bacterales bacterium*	0.034±0.13	0.0099±0.041	0.8336	-0.024±0.12	0.0052±0.027	0.017±0.092	0.2084	0.012±0.065	0.5457
	Erysipelotrichaceae UCG-003	0.20±0.32	0.16±0.31	0.2993	-0.034±0.34	0.25±0.48	0.75±3.55	0.8287	0.50±3.46	0.6074
	Gemella	0.0038± 0.0077	0.0047± 0.0080	0.5135	0.00094± 0.0073	0.014± 0.054	0.0044± 0.010	0.1153	-0.0096± 0.054	<0.05
	Dorea	0.27±0.25	0.28±0.42	0.3528	0.012±0.47	0.47±0.77	0.38±0.69	0.1874	-0.093±1.012	0.9130
	Lachnoclostridium	1.067±1.49	0.90±1.20	0.6762	-0.16±1.49	0.94±1.39	1.15±1.20	0.2833	0.22±1.63	0.1589
	Lachnospira	0.74±0.90	0.56±0.81	0.2665	-0.17±0.87	0.56±0.62	0.77±0.98	0.6202	0.21±1.060	0.3310
	Lachnospiraceae UCG-006	0.27±0.36	0.25±0.41	0.9033	-0.022±0.49	0.22±0.54	0.39±0.72	0.0655	0.17±0.43	0.1656
	Lachnospiraceae UCG-010	0.12±0.26	0.12±0.20	0.9508	-0.0067±0.26	0.12±0.15	0.15±0.21	0.2907	0.032±0.23	0.3847
	Roseburia	1.041±1.064	0.83±1.14	0.4175	-0.21±1.11	0.79±0.98	1.076±1.54	0.1689	0.29±1.80	0.06658
	[Eubacterium] eligens group	0.92±1.22	0.82±1.28	0.8523	-0.098±1.40	0.44±0.73	0.67±1.24	0.5027	0.23±1.46	0.4644
	[Eubacterium] ruminantium group	0.34±0.78	0.32±0.68	0.9839	-0.015±0.96	0.23±0.55	0.30±0.79	0.6603	0.072±0.72	0.9067
	[Eubacterium] xylanophilum group	0.038±0.14	0.029±0.089	0.9057	-0.0090±0.16	0.028±0.086	0.033±0.090	0.7837	0.0048±0.078	0.5541

	[Ruminococcus] gauvreauii group	0.039±0.069	0.030±0.058	0.3411	-0.0093± 0.037	0.021±0.036	0.065±0.21	0.2180	0.044±0.21	0.2352
	Unclassified Lachnospiraceae	2.71±2.83	2.01±2.14	0.2689	-0.70±3.44	1.32±1.59	1.93±2.17	0.1118	0.61±1.98	0.05850
	Lactobacillus	0.18±0.62	0.048±0.13	<0.05	-0.13±0.51	0.023±0.066	0.024±0.064	0.2425	0.00047± 0.048	<0.01
	Weissella	0.075±0.36	0.011±0.023	0.1280	-0.064±0.36	0.0068± 0.0085	0.058±0.13	0.4237	0.051±0.13	<0.05
	Romboutsia	0.15±0.35	0.094±0.16	0.6043	-0.058±0.32	0.11±0.20	0.14±0.23	0.4270	0.030±0.23	0.3033
	Butyricicoccus	0.14±0.25	0.19±0.24	0.2603	0.050±0.31	0.12±0.20	0.12±0.14	0.5987	-0.0014±0.24	0.6008
	Flavonifractor	0.13±0.24	0.094±0.18	0.1415	-0.036±0.16	0.18±0.33	0.20±0.37	0.7003	0.012±0.36	0.4758
	Ruminococcaceae NK4A214 group	0.18±0.17	0.20±0.22	0.6903	0.012±0.21	0.20±0.37	0.26±1.033	0.2427	0.060±0.86	0.6641
	Unclassified Ruminococcaceae	0.26±0.75	0.25±1.068	0.8108	-0.0082±0.56	0.068±0.099	0.072±0.17	0.5115	0.0045±0.14	0.8180
	Lactococcus	0.0088± 0.046	0.00091± 0.0027	0.3590	-0.0079± 0.047	0.0021± 0.0077	0.052±0.15	<0.01	0.050±0.15	<0.01
Fusobacteria	*Fusobacterium*	0.24±1.37	0.50±2.94	0.5839	0.27±1.58	0.37±1.88	0.20±0.87	0.4148	-0.17±1.041	0.2931
Proteobacteria	*Sutterella*	0.99±1.30	0.80±1.38	0.5306	-0.19±1.34	0.87±1.42	1.43±2.12	<0.05	0.56±1.36	<0.05
	Escherichia-Shigella	0.29±0.65	1.68±4.89	0.5370	1.38±4.91	0.60±1.96	0.32±1.072	0.1551	-0.28±0.95	0.2720

Baseline, Week 0; Endline, Week 8. p^a, *p*-value for paired t-test or Wilcoxon signed rank test compared within placebo group; p^b, *p*-value for paired t-test or Wilcoxon signed rank test compared within HDB1258 group; p^c, *p*-value for two sample *t*-test or Wilcoxon rank sum test compared between groups for changed value.
